# Loki zupa alleviates inflammatory and fibrotic responses in cigarette smoke induced rat model of chronic obstructive pulmonary disease

**DOI:** 10.1186/s13020-020-00373-3

**Published:** 2020-08-31

**Authors:** Nabijan Mohammadtursun, Qiuping Li, Muhammadjan Abuduwaki, Shan Jiang, Hu Zhang, Jing Sun, Jingcheng Dong

**Affiliations:** 1 Department of Integrative Medicine, Huashan Hospital, Fudan University, Shanghai, 200040 China; 2 College of Xinjiang Uyghur Medicine, Hotan, China

**Keywords:** Loki zupa formula, Traditional medicine, Cigarette smoke, COPD, Gene expression profile

## Abstract

**Background:**

Loki zupa formula is kind of a traditional medicines which used to treat airway diseases, especially those caused by abnormal phlegm, such as cough, asthma and chronic bronchitis. The study aim was to explore the anti-inflammatory and anti-remodeling effects of Loki zupa by using a cigarette-smoke induced rat model of chronic obstructive pulmonary disease.

**Methods:**

The rats were divided into five groups: the normal group, the model group, the LZ 4 g/kg and LZ8g/kg group, and the positive control group. Rats were exposed to cigarette smoke for 24 weeks to induce a COPD rat model. Lung function was assessed. Histopathological changes were recorded using Haematoxylin–eosin and Masson’s trichrome staining. Mucus hypersecretion was evaluated by PAS staining. Inflammatory factors were measured in blood serum and bronchial alveolar lavage fluid using an enzyme-linked immunosorbent assay. Malondialdehyde and superoxide dismutase and glutathione *S*-transferase levels were tested by biochemical methods. Gene expression patterns were evaluated using GN-GeneChip Clariom S Array for rat from Affymetrix. And top upregulated and downregulated genes validated by qPCR. And these genes was also compared with gene transcriptomic data from smoker patients with emphysema and non-smokers in GEO dataset. IL-6/PLAGA2A signalling protein expression was assessed by western blot and immunohistochemistry. TGF-β1and smad2/3 signalling expressions were analysed by western Blot.

**Results:**

Loki zupa improved COPD rats lung function as compared to the model group and pathological changes including inflammatory cell infiltration and goblet cell metaplasia was alleviated in rats treated with Loki zupa Inflammatory factors IL-6, TNF-α, IL-1β and TGF-β1 decreased while significant increase was observed in blood serum IL-10 content in rats treated with Loki zupa. And IL-6 and TNF-α level in bronchial alveolar lavage fluid showed same expression trend in blood serum, while there was no change in MMP-9 content. It also increased antioxidant enzyme SOD and GPX activity while reducing the lipid peroxidation. Gene microarray analysis showed that there were 355 differentially expressed gene in LZ treated COPD rat lung as compared to model group. Both microarray and qPCR results showed that top differentially expressed genes nxt1 (up regulated) and pla2g2a (down regulated) expression were also reversed by LZ treatment. And protein expression level of IL-6 and pla2g2a was also elevated in CS exposed rats while significant reduction was observed in LZ treated rats. Accordingly, Loki zupa inhibited Collagen-1 upstream protein expression of TGF-β/smad2/3 signalling pathway.

**Conclusion:**

These results demonstrated that Loki zupa showed protective effects in the lung of the COPD rat model. This mainly because of Loki zupa exerts anti-inflammatory effects by blocking IL-6/pla2g2a signalling and inhibiting inflammatory gene expression and attenuates fibrotic responses by inhibiting TGF-β/smad2/3 signalling pathway.

## Background

Chronic obstructive pulmonary disease (COPD) is a combination of small airway diseases like obstructive bronchiolitis and parenchymal destruction which causes chronic airflow limitation [1]. It is one of the most burdensome and costly chronic illnesses in the world. Globally, COPD accounted for 2.9 million deaths in 2016 alone [2]. The global cost for COPD in 2012 was approximately $5.2 billion, and increased annually with a 5-year compound rate of 6% [3]. Understanding of the pathogenesis and optimal treatment is still incomplete because multiple factors contribute to the pathogenesis of COPD. Usually, chronic persistent inflammation is activated by significant exposure to noxious particles or gases such as cigarette smoke [4]. Oxidative stress is a significant characteristic of pulmonary inflammation and also contributes to the amplification of inflammatory responses in patients with COPD [5]. However, while there is no effective cure, quitting smoking is the single best option for reducing development and progression of the disease [6–8]. Although there have been improvements in the management of COPD, nonetheless, there is still a huge unmet need to find medical therapies which decrease disease prognosis and death rate.

As COPD is the result of complex chronic inflammation of the airways; the most direct medical interventions target inflammation. When the airway is stimulated by external irritants, an innate immune response is initiated and then mediates the processes of inflammation, repair, fibrosis and proteolysis [9, 10]. Oxidative stress also activates the pro-inflammatory factors, such as chemokines, TNF-α, IL-1β, IL-6, MMPs, and TGF-β. Altogether, they initiate neutrophilic inflammation, the destruction of lung parenchyma, and airway fibrosis [11]. As a pro-inflammatory cytokine, interleukin 6 (IL-6), is implicated in a variety of inflammatory diseases [16] including COPD and athsma. PLA2G2A (type-II secretory phospholipase A2/sPLA2), an enzyme associated with acute respiratory distress syndrome (ARDS), PLA2G2A expression was increased under both IL-6 and IL-6 trans-signalling conditions and amplified inflammatory responses in human airway smooth muscle cells [12]. It was also reported that LPS induces increased PLA2G2A expression and secretion in lung epithelial cells [13]. sPLA2 is also a novel invasion-promoting gene in lung adenocarcinoma and can predict the clinical outcome of lung adenocarcinoma patients [14]. however, the effects of IL-6- PLA2G2A signalling, and PLA2G2A expression in COPD have not been investigated. We also hypothesized that blocking IL-6-PLA2G2A signalling may contribute to reduction of inflammatory responses and progression of the disease.

TGF-β1is one of the distinct fibrotic factors, playing an important role in the airway remodeling in COPD [15]. TGF-β1transduces fibrotic signals to intracellular Smads and results in the phosphorylation of smad2/3. This increases inflammation and ECM production in lung tissue. Up regulation of TGF-β ligands is observed in major pulmonary diseases including COPD [16]. Thus, blocking TGF-β1/smad2/3 signalling may contribute to reduce fibrotic signals.

loki zupa is traditional medicine which composed of *Hyssopus cuspidatus* Boriss. (shen xiang cao) and *Iris halophila* Pall. (Yuanweigen) used to treat airway diseases caused by abnormal phlegm such as cough, bronchitis, dyspnea [17], especially among the ethnic Uyghur population in the Xinjiang region of China. Our previous studies showed that Loki zupa water extract reduces airway inflammation in an asthmatic murine model [18]. A multi-centered, double blinded and randomized clinical trial also revealed Loki zupa is effective for treating asthma [19].There is a report that ethanol extracts of on *H. cuspidatus* prevented bronchial asthma by inhibiting MAPK/NF-κB inflammatory signaling [20]. And its chemical components exerts potent antioxidant capacity [21]. And *H. cuspidatus* revealed an anti-inflammatory effects by restoring Th1/Th2, Th17/Treg balance in murine model of COPD [22]. And *I. halophila* Pall. also used as antispasmodic drug in traditional Uyghur medicine. These results indicated the possible role of Loki zupa in treating COPD. However, the effects and mechanisms of Loki zupa on COPD have not yet been evaluated. Thus, in this study we used cigarette smoke-induced COPD rat model and evaluated its effects on airway inflammation and airway-remodeling in the model.

## Methods

### Preparation of Loki zupa extract

Decoction pieces of *H. cuspidatus* Boriss. (batch number: C30196901) and *I. halophila* Pall. (batch number: G30009407) was purchased from Xinjiang Maidisen traditional Uyghur medicine Co. Ltd.(Hotan, China) and identified as pure and eligible medicinal materials for further use. Dried *H. cuspidatus* Boriss. and *I. halophila* Pall. was mixed in a ratio of 2:1 (900 g:450 g) and immersed for 1 h with 10 times water. They were then boiled twice for an hour. The extracts was collected and filtered, and then concentrated to 4 g/ml. They were kept in sterile bottles and stored at 4℃. Loki zupa extract was intragastrically administered to rats in final concentrations of 4 g/kg and 8 g/kg.

### Reagents and chemicals

Dexamethasone sodium phosphate (Dex) was provided by Huashan hospital affiliated to Fudan University. 3R4F research cigarettes were purchased from the University of Kentucky (Lexington, KY, USA) and chloral hydrate was purchased from Aladdin (Shanghai, China). IL-10, IL-6, TNF-α, and IL-1β, MMP-9 TGF-β enzyme-linked immuno-absorbent assay (ELISA) kits were purchased from Raybiotech. (Norcross, USA), SOD, GPX, MDA assay kits was purchased from BioTNT (Shanghai, China), Anti-sPLA2 (ab23705), Anti-IL-6 (ab9324), Anti-Collagen-1 antibody (ab90395) was purchased from Abcam., Anti-TGF-β1 (#3709), anti-smad2/3 (#8685), anti-psmad-2/3 (#8828), and β-actin (#3700) antibodies were produced by Cell Signaling Technology (Danvers, USA). Acetonitrile (pubchem CID: 6342), formic acid (pubchem CID: 284). Reference standards luteolin(Pubchem CID: 5280445), apigenin (Pubchem CID: 5280443), luteolin-7-*O*-β-glucoside (pubchem CID: 5280637), apigenin-7-*O*-β-glucoside (pubchem CID: 12304093), Diosmetin-7-*O*-β-glucopyranoside(pubchem, CID: 11016019), rosmarinic acid (pubchem CID:5281792), Chrysin-7-*O*-β-glucoronide (pubchem CID:14135334) were purchased from Shanghai Winherb Medical & development Co.Ltd(Shanghai, China).

### Chromatograph and mass condition

Agilent series 1260 HPLC instrument equipped with a binary pump, a diode-array detector, an auto-sampler, and a column compartment (Agilent, Waldbronn, Germany), and 6530 Q-TOF mass spectrometer (Agilent, Technologies, Santa Clara, CA) via an ESI interface was used to analyze the active constituents. For the nebulizing and collision gases, High-purity N2 and ultrahigh purity He were used respectively. The experiment was performed on mass spectrometer source in negative ion mode and for qualitative analysis, operated in auto MS/MS scan mode, the parameters were set as follows: end plate offset -500 V, capillary voltage, 3500 V; temperature, 350℃ and flow rate was 10 l/min; nebulizing gas, 30 psi; fragmentor, 125 V; collision energy, 20 eV; molecular weight scanning range was 100–1500 Da. All data were acquired and processed Mass Hunter Workstation Qualitative Analysis Software (Version B. 06.00, Agilent Technologies). The mobile phase was consists of water containing 0.1% v/v acetonitrile (A) and formic acid (B) at flow rate of 0.35 ml/min.

### Animals and treatments

40 male Sprague–Dawley (SD) rats, 5–6 week old, weighing 100 ± 20 g, were purchased from Xipuer–Bikai Laboratory Animal Co., Ltd. (Shanghai, China; license number, SCXK [Hu]2008-0016). All the rats (4 rats/cage) were placed in a specific pathogen-free (SPF) laboratory in the Animal Center of Shanghai Medical College of Fudan University, at 22 ± 1 °C temperature and 50 ± 5% humidity under a 12 h light/dark cycle with food and water ad labium. All experimental protocol were performed in accordance with the National Institutes of Health Guidelines for the Care and Use of Laboratory Animals. Procedures involving animals and their care were conducted carefully and treated with minimum pain.

### Experiment protocols

After one week accommodation, the rats were divided into the following five groups: the normal group (RA), the CS exposure group (CS), the Loki zupa 4 g/kg group (LZ-4), the Loki zupa 8 g/kg group (LZ-8), and texamethasone group (Dex), and 8 rats in each group. The normal group was exposed to surrounding air in the room. All the other groups were exposed to side-stream cigarette smoke generated from Buxco smoke generator (Buxco, NC, USA) by using3R4F cigarettes for 6 days a week. Briefly, rats were placed into the self-made plastic smoking chamber (100 * 70 * 40) and 12 cigarettes were used to generate smoke and rats were exposed to the cigarette smoke once a day, 6 days a week. The CS exposure period was 24 weeks. From the first week of exposure, the rats in dexamethasone (Dex) group were treated with 0.1 mg/kg/day dexamethasone. The rats in low and high dose group of Loki zupa were treated with 4 g/kg/ day and 8 g/kg/ day. Simultaneously, the rats in room air and model group were intragastrically treated with the same amount of saline water. Body weight was measured weekly to adjust drug administration level and relevant indicators were tested. Before sample collection and lung function analysis, rats were anesthetized by intraperitoneal injection of 40 mg/kg, 10% chloral hydrate. After sample collection, remained body of rats was treated according to the injurious medical waste disposal procedure.

### Lung function

After anesthetized, each rat was tracheostomized and placed in a lung function detection chamber which was connected to a computer (Buxco, NY, USA) and forced to an average breathing frequency of 150 breaths/min. Functional residual capacity (FRC) was determined by forcing rats to breathe against a closed valve at the mouth. The lungs were inflated by a standard pressure of + 30 cmH_2_O. Different lung volumes, such as forced vital capacity (FVC) and the forced expiratory volume in 0.1 s (FEV0.1), peak expiratory flow (PEF), mid-maximal expiratory flow (MMEF) were then recorded by the pulmonary function maneuver system.

### Sample collection

Just after lung function analysis, samples were collected from the anesthetized rats. Blood samples were harvested from the abdominal artery and placed in ice for 2 h prior to centrifugation. Blood was centrifuged at 3000 rpm for 30 min to obtain serum samples. The supernatant was obtained and stored at − 80 °C. Bronchoalveolar lavage fluid was harvested as previously described [23, 24] and stored at − 80 °C for the cytokines assay. The right lung was removed and placed in liquid nitrogen for 30 min and then stored at − 80 °C for further analysis. Death was confirmed after blood and lung sample collection.

### Histology and immunohitochemistry

The right lung was harvested for histopathological analysis, fixed with 4% formaldehyde and paraffin-embedded, sliced into 4 um thick sections, positioned on poly-llysine-coated slides, and then incubated at 58 °C for 24 h. Deparaffinized sections were stained with hematoxylin and eosin (H&E) and followed by staining with Masson’s trichrome and periodic acid–Schiff (PAS). Immunohistochemical analysis was performed on 3 µm formalin fixed sections. PL2AG2 immunohistochemistry were performed as previously describe. Using anti-secretory phospholipase A2 antibody and anti-IL-6 antibody.

### Quantification of cytokines

Interleukin-6 (IL-6), Interleukin 1β (IL-1β), Interleukin-10 (IL-10), tumor necrosis factor-α (TNF-α), transforming growth factors-β (TGF-β) in the blood serum, and Interleukin-6 (IL-6) and Matrix metalloprotein-9 (MMP-9) were quantified in BALF from various treatment groups by ELISA according to the manufacturer’s protocol.

### Antioxidant detection

The levels of superoxide dismutase (SOD), glutathione *S*-transferase (GSH-ST), glutathione peroxidase (GPX) and malondialdehyde (MDA) content in serum were determined using biochemical kits (BioTNT Co.Ltd.) following the manufacturer’s instructions.

### Gene expression profile and bioinformatics

The total RNA of the lung tissue were extracted and purified, using miRNeasy mini kit (Qiagen, Germany) according to the manufacturer's instructions. Quantitation and quality assessment of the RNA were assessed by using Thremo Nanodrop 2000 (Thermo, USA) and the Agilent 2100 bio-analyzer and the Agilent RNA 6000 Nano Kit (Agilent Technologies, Palo Alto, Calif), respectively. Only RNA samples with an optical density 1.7 < A260/A280 < 2.2, as well as bio-analyzer results RIN ≧ 7.0 and 28S/18S > 0.7 were used in further experiments. cDNA was run on Clariom S Array for rat (Affymetrix, USA) following the manufacturer protocol. Affymetrix GeneChip Scanner 3000 (Affymetrix, USA) was used for signal detection. Expression Console soft-ware (Affymetrix, USA) was used to check the quality.

### qPCR analysis

To further validate differential mRNA expression from lung tissues in the COPD rats, the total RNA was extracted form lung tissue using trizol reagent and RNA was converted to cDNA using PrimeScript™ RT Master Mix (Takara). Quantitative PCR (qPCR) was performed using TB green premix Ex Taq II (Takara), according to the manufacturer's protocol, cDNA samples, appropriate primers, and TB Green Premix Ex Taq II (Takara), ROX Reference Dye (50×, ddH2O. Primers used for qPCR were as follows: as follows: NXT1, forward 5-CTTTGTCAGCTCCGTCTTCA-3, reverse5-CTCAAGGCCTTCTCCAGT TC-3; PLA2G2A, forward 5-GCACA GTTGGCAACCTTTATG-3, reverse 5-CCATCAGCATCATACACTCCTC3; GAPDH, forward 5-AACTCCCATTC CTCCACCTT -3, reverse 5-GAGGGCCTCTCTCTTGCTCT-3. The thermal cycling conditions included initial incubation of samples at 95 C for 30 s, followed by appropriate cycles of 95 C for 5 s, 60 C for 30 s. GAPDH used as an internal control, and relative expression levels were determined using the 2 − ΔΔCt method.

### Western blot analysis

The right lung in each group was harvested and total protein was obtained and quantified by a BCA procedure. The isolated total proteins were separated by SDS-PAGE electrophoresis. And then transferred onto PVDF membranes. The membranes were blocked with 5% skim milk in Tris buffer saline-Tween20 (TBST) and washed three times for 5 min each time. They were then incubated overnight at 4℃ with their respective antibodies. After washed with TBST three times for 15 min, the membranes were incubated with a horseradish peroxidase conjugated secondary antibody (1:10,000) for 1 h at room temperature. The membranes were then visualized using the enhanced chemiluminescence detection system (Pierce, USA). The level of β-actin was used as an internal control. Relative intensities were quantified using Quantity One (Bio Rad).

### Statistical analysis

The data is expressed as the means ± SDs and was analyzed using one-way analysis of variance (ANOVA) with the turkey multiple comparison test. A p value < 0.05 was considered significant.

## Results

### Qualitative analysis of bioactive compounds in Loki zupa

**Fig. 1:**
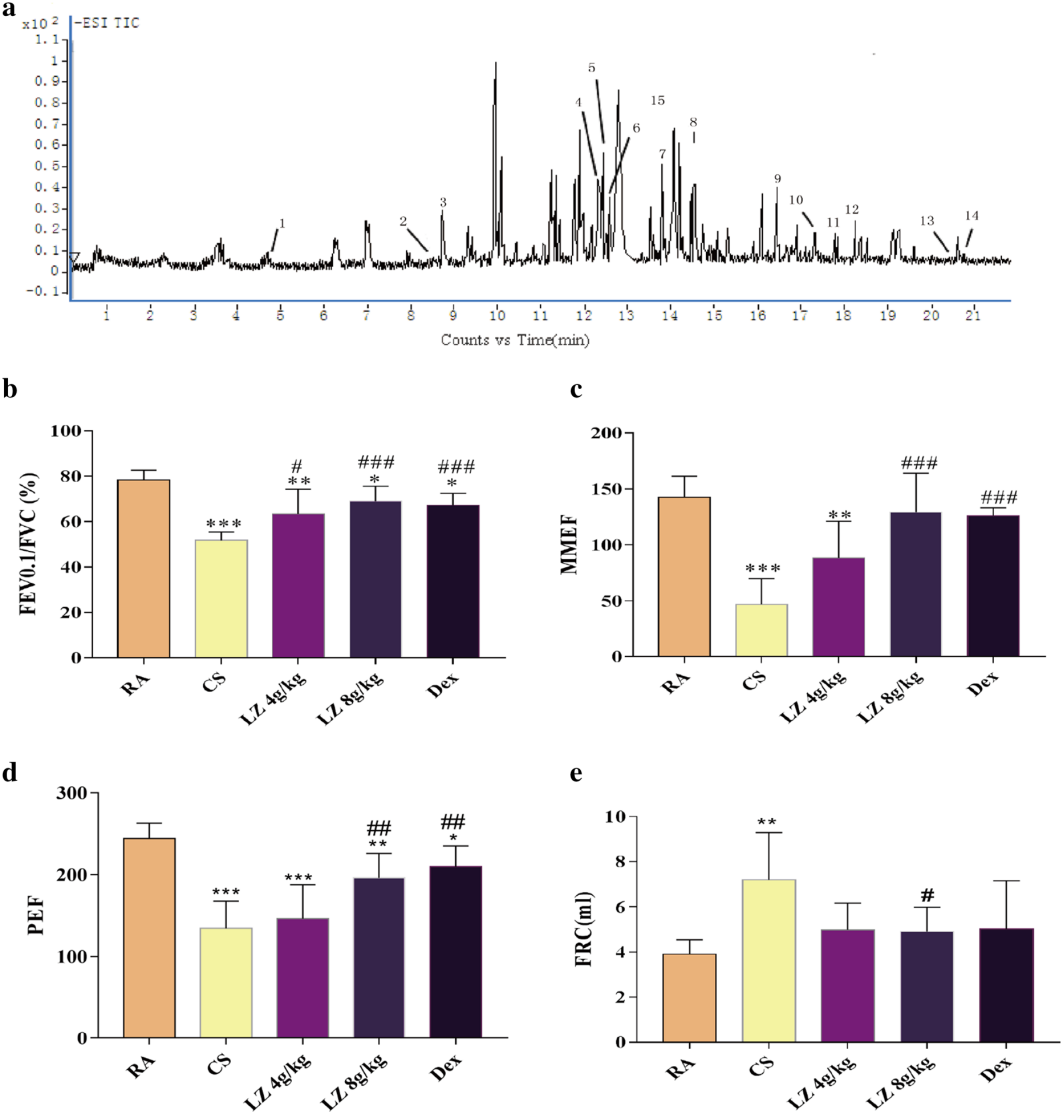
LC-ESI-Q/TOF-MS characteristic chromatogram of LZ formula, lung function parameters. A.Total ion chromatograms (TIC) of Looki zoofa sample[1: caffeic acid methyl este; 2: Tectorigenin A; 3: Iristectorin A; 4: Luteolin-7-*O*-β-d-glucuronide; 5: Luteolin-7-*O*-β-d-glucoside; 6: Iristectorigenin A-7-*O*-β-glucoside; 7: Iristectorin A; 8: rosmarinic acid; 9: Luteolin;10: Acacetin-7-*O*-β-glucuronide; 11: Apigenin; 12: Iristectorigenin B; 13: Irilone a; 14: 5,7-dihydroxy- 6,4′-dimethoxyisoflavone. B.FEV0.1 s/FVC; C.MMEF; D. PEF; E. FRC. Data expressed as means ± SD. Compared with control: *p < 0.05, **p < 0.01, ***p < 0.001 Compared with model: ^#^p < 0.05, ^##^p < 0.01, ^###^p < 0.001

Liquid chromatography coupled to quadruple time-of-flight with tandem mass spectrometry (LC-ESI-Q/TOF–MS) method was used to identify chemical constituents of Loki zupa. A representative total ion chromatogram was shown in Fig. 1a. Loki zupa formula contains wide range of compounds which including caffeic acid methyl ester, Tectorigenin A, Iristectorin A, Luteolin-7-*O*-β-d-glucuronide, Luteolin-7-*O*-β- d -glucoside, Iristectorigenin A-7-*O*-β-glucoside, Iristectorin A, rosmarinic acid, Luteolin, Acacetin-7-*O*-β-glucunide, Apigenin, Iristectorigenin, Irilone A, 5,7-dihydroxy-6,4′-dimethoxyisoflavone. Among which luteolin and apigenin have been proven to be effective treating airway disease and lung cancer.

### Effects of LZ on lung function

After 24 weeks of CS exposure, COPD rats displays significantly declined FEV_0.1 s_/FVC, MMEF, PEF and increased FRC value as compared to the control group (p < 0.001, Fig. 1b–e). Indicating that COPD model establishment was successful. After treatment with both low and high doses of LZ, the FEV_0.1 s_/FVC ratio increased dramatically in different levels in contrast to the CS group (p < 0.05, p < 0.001, Fig. 1b). There was no significant change after treatment with LZ 4.0 g/kg, but a high dose of LZ did increase MMEF and PEF (p < 0.01 and p < 0.001, Fig. 1c, d). FRC was increased in CS exposed rats (p < 0.01) and reversed after 8 g/kg LZ treatment rats (p < 0.05, Fig. 1e). The Dex treated group showed increased MMEF and FEV0.1/FVC ratio as compared to that of the CS group (p < 0.01).

### Effects of LZ on inflammatory cell infiltration and mucus hypersecretion

**Fig. 2:**
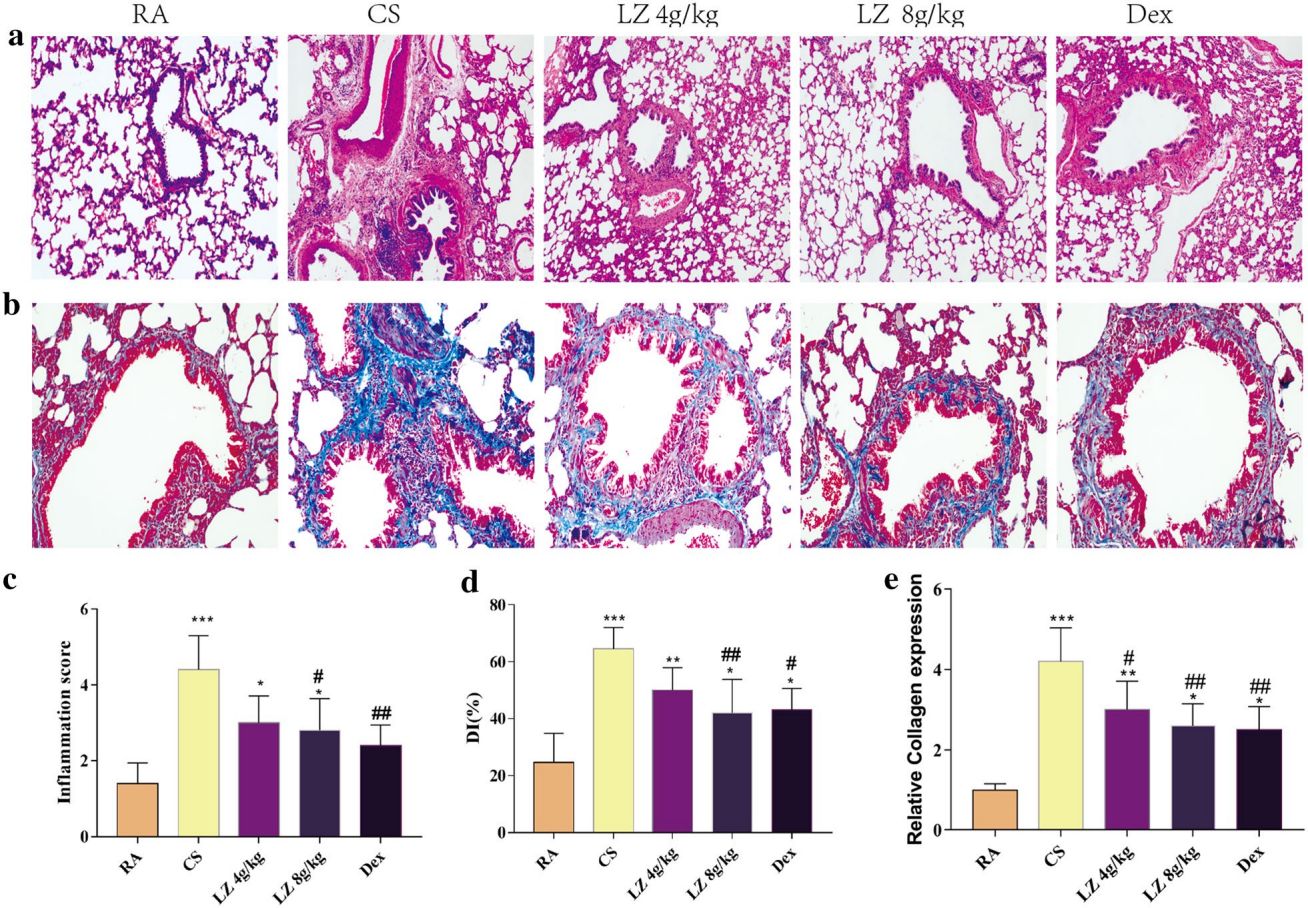
Effects of LZ on inflammatory cell infiltration. **a** H&E staining(40×); **b** Masson’s trichrome staining(100×); **c** inflammation score; **d** destructive index; **e** relative collagen deposition. Data expressed as means ± SD. Compared with control: *p < 0.05, **p < 0.01, ***p < 0.001 Compared with model: ^#^p < 0.05, ^##^p < 0.01, ^###^p < 0.001

As revealed in Fig. 2, rats’ lung in COPD group had higher airspace diameter and obvious inflammatory cells infiltration as compared to RA group. However, corresponding phenomena was reversed in LZ treated groups at a different level than the model group (Fig. 2a). Inflammation scores and alveolar destructive index was also evaluated, results showed that lung of COPD rats had a higher inflammation score and destructive index than those in normal group (p < 0.001, Fig. 2c, d). However both inflammation score and destructive index decreased after treatment with LZ to a different extent (p < 0.05, p < 0.01, respectively).

To examine the protective effect of LZ on CS-induced fibrosis in COPD rat lung, Masson's trichrome staining for collagen was applied. In the lung tissue from CS group rats, extensive collagen staining was observed around airway (Fig. 2b, p < 0.001). In rats treated with LZ, However, collaged deposition in lung tissue was markedly reduced as compared to the CS group (p < 0.05, Fig. 2b, e).

**Fig. 3:**
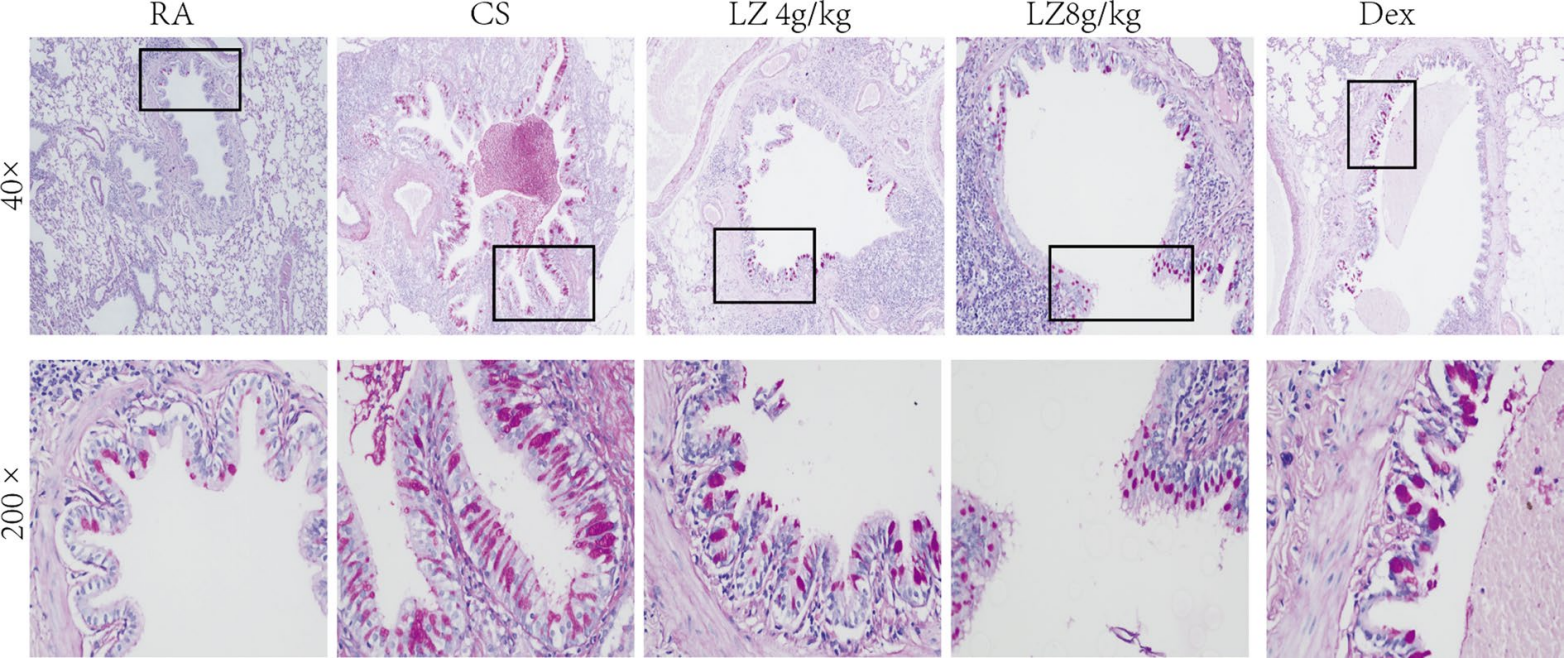
Effects of LZ on mucus hypersecretion by PAS staining. Data expressed as means ± SD. Compared with control: *p < 0.05, **p < 0.01, ***p < 0.001 Compared with model: ^#^p < 0.05, ^##^p < 0.01,^####^p < 0.001

Goblet products in epitheliums of trachea were stained by PAS, and then a positive PAS-stained area was measured and analyzed as goblet metaplasia and mucus secretion. According to histological evaluation, goblet cell proliferation and mucus occlusion induced by cigarette smoke were significantly increased in the CS induced rat model as compared to that of the RA group (p < 0.001, Fig. 3). However, rats treated in the LZ (4 g/kg and 8 g/kg) groups exhibited reduced levels of mucous secretion and the decreased goblet cell proliferation (PAS+ cells) compared to that of the COPD rats (p < 0.001, Fig. 3).

### Effects of LZ on pro-inflammatory and anti-inflammatory factors in blood serum

**Fig. 4:**
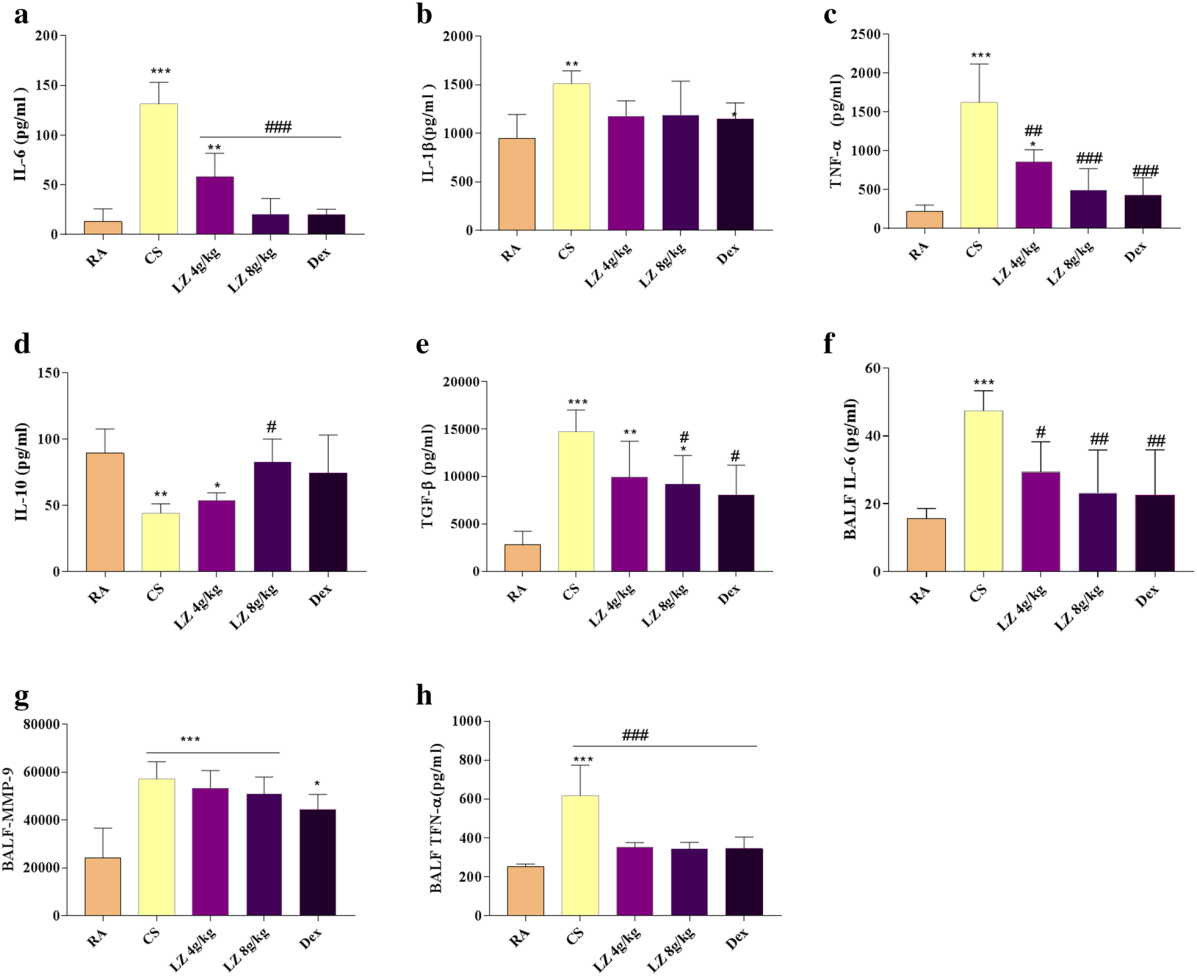
Effects of LZ on pro-inflammatory and anti-inflammatory factors, antioxidants and lipid peroxidation indicators in blood serum, **a**: IL-6; **b** IL-1β; **c** TNF-α; **d** IL-10; **e** TGF-β1

**Fig. 5:**
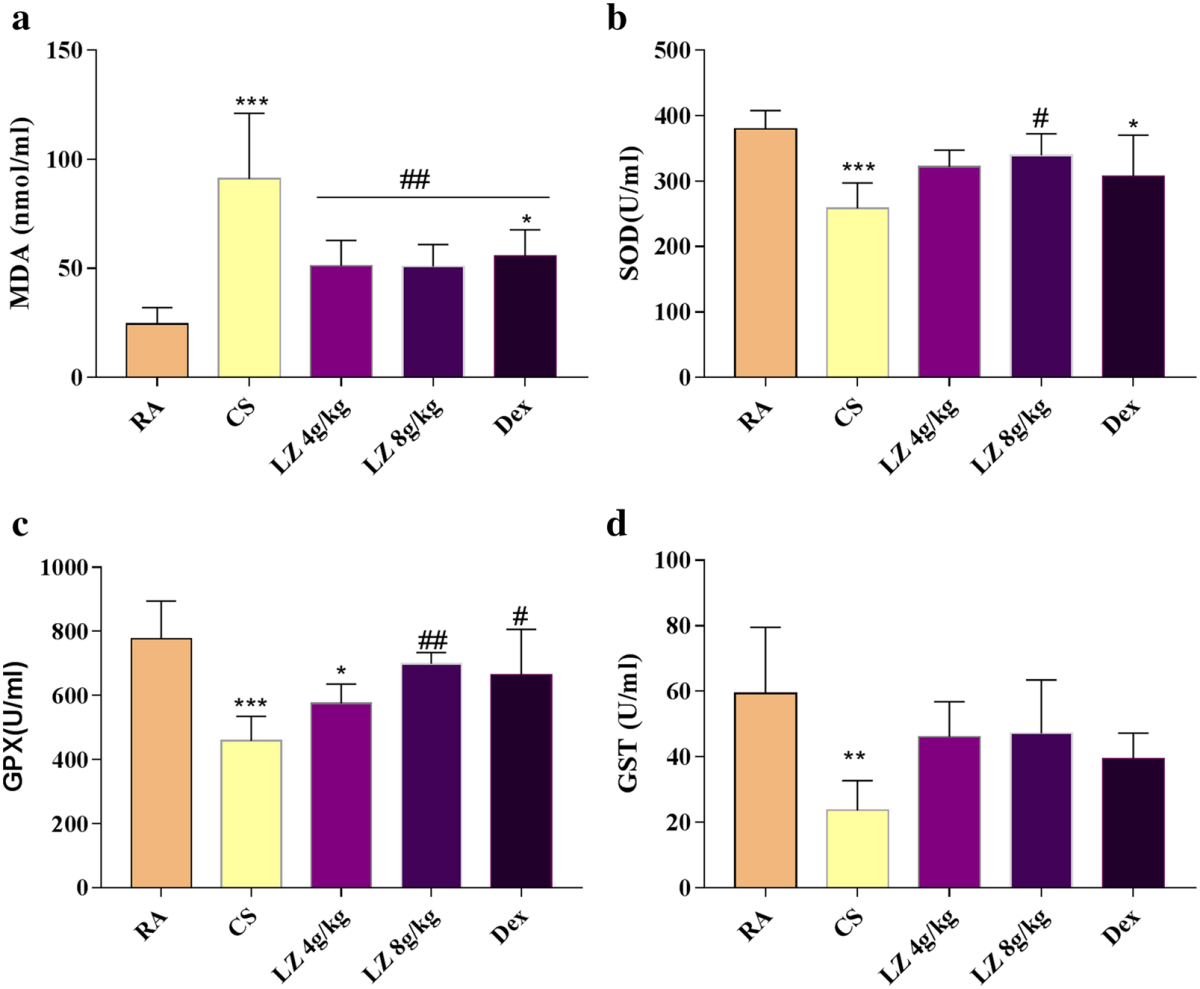
Effects of LZ on lipid peroxide peroxidation and antioxidants in COPD rat model. **a** MDA; **b** SOD; **c** GPX; **d** GST. Data expressed as means ± SD. Compared with control: *p < 0.05, **p < 0.01, ***p < 0.001 Compared with model: ^#^p < 0.05, ^##^p < 0.01, ^###^p < 0.001

Next, we tested main pro-inflammatory and anti-inflammatory factors in the blood serum, As shown in Fig. 5, compared with the RA group, IL-6 and TNF-α levels in blood serum sharply increased after 24 wk CS exposure (p < 0.001). However, both LZ treatment groups and Dex group showed reduced IL-6 level than that of the CS group (p < 0.001, Fig. 4a). TNF-α level also significantly decreased in the LZ 8 g/kg and Dex groups (p < 0.01, Fig. 4c). Increased IL-β, TGF-β1 levels were observed in the CS exposure group compared with those rats in the RA group (p < 0.05, Fig. 5b) while IL-10 level reduced significantly (p < 0.05, Fig. 4d). Both low and high dose LZ treatment groups did not reduce increased IL-1β level as compared to CS group (p > 0.05), and there was obvious reduction in TGF-β1 concentration after treated with 8 g/kg LZ and Dex (p < 0.05, p < 0.01, Fig. 4e). Significant increase was observed in anti-inflammatory factor IL-10 levels after treatment with LZ-8 g/kg (p < 0.05, Fig. 4d) while there was no change in LZ-4 g/kg and Dex groups as compared to the CS group (p > 0.05).

Inflammatory factors in BALF were also measured. As shown in Fig. 4, IL-6, MMP-9 and TNF-α concentration increased in CS group (p < 0.001) and Both IL-6 and TNF-α content were markedly decreased after LZ treatment (p < 0.05, p < 0.01, p < 0.001, respectively, Fig. 4f, h). And as compared to model group, there was no significant change in BALF MMP-9 level in LZ treatment group (p > 0.05, Fig. 4g).

### Effects of LZ on lipid per oxidation and antioxidants

Lipid peroxidation level was observed by measuring MDA content. Compared with RA group, COPD rats had higher MDA content in blood serum (p < 0.001). However, rats in LZ treated groups and Dex groups displayed reduced MDA level as compared to CS groups (p < 0.01, Fig. 5a). Next, we determined SOD, GST and GPX contents, In comparison with the RA group, SOD, GPX and GST content declined in CS group (p < 0.01, Fig. 5b, d). However, SOD and GPX level significantly elevated after 8 g/kg LZ treatment (p < 0.05, p < 0.01 respectively, Fig. 5b, c). And there was an increasing trend in GST activity but statistically no significant as compared to model group (p > 0.05, Fig. 5d).

### Effects of LZ on gene expression profiles in lung of CS induced COPD rats

**Fig. 6:**
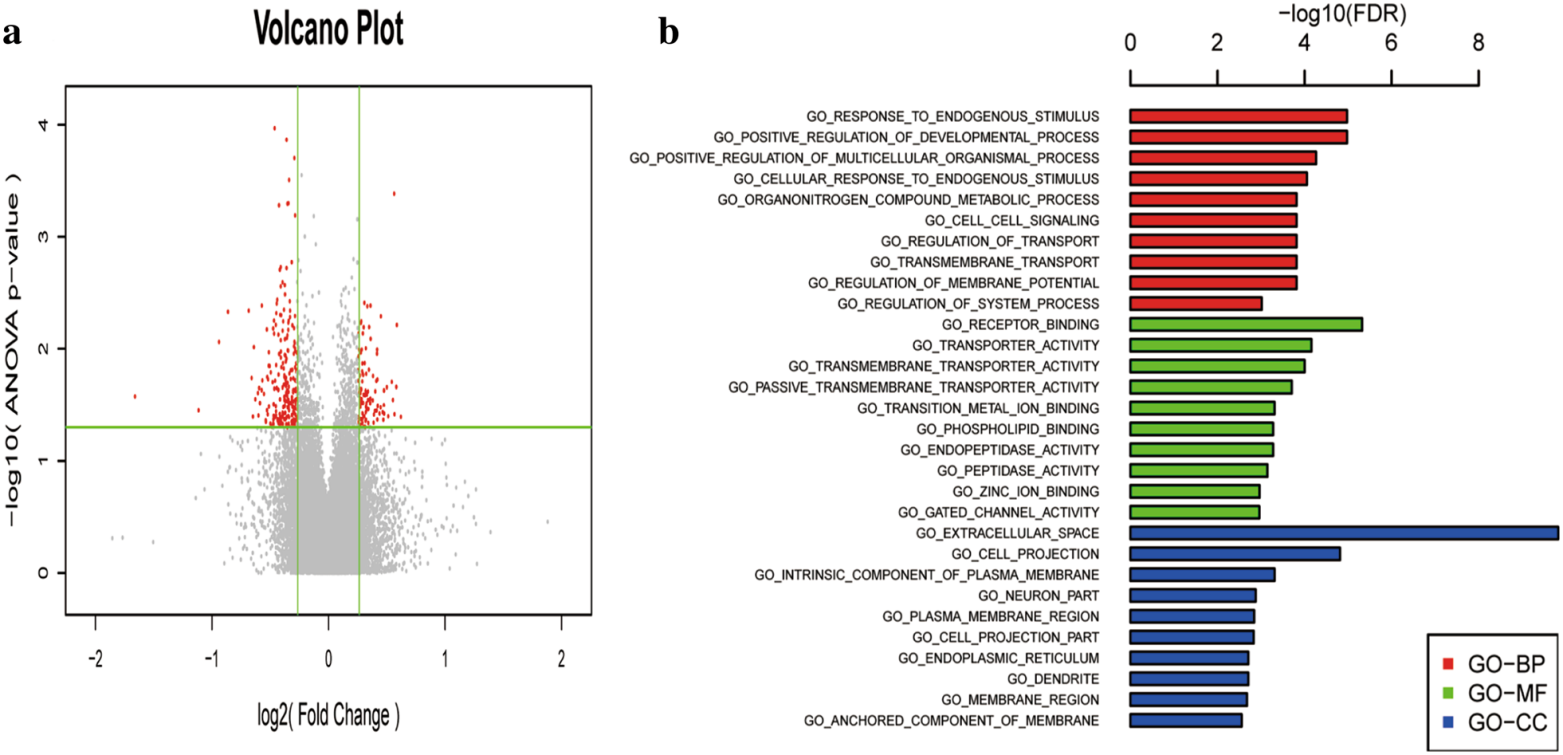
**a** Volcano Plot. **b** Gene ontology analysis of differentially expressed proteins between CS group and LZ treated group

Gene microarray analysis of lung samples from CS induced COPD rats displayed significantly up-regulated-expression of (> 1.2-fold) 232 genes and decreased expression of (>− 1.2 fold) 135 genes (Fig. 6a, Additional file 1: Figure S1) and top 10 upregulated and down regulated genes by LZ treatment given in Table 1. Gene ontology analysis of LZ modulated genes also suggested significant effect on genes associated with biological processes such as positive regulation developmental process, response to endogenous stimulus, transmembrane transport, regulation of membrane potential, et al. Molecular function including receptor binding, transport activity, phospholipid binding. Cellular components comprising extracellular space, intrinsic component of plasma membrane, neuron part, cell projection part (Fig. 6b).

**Table 1:** Top 10 upregulated and downregulated genes by loki zupa

NO	Gene Symbol	Description	Fold change	p-value	Entrez ID	Expression
1	Nxt1	Nuclear transport factor 2-like export factor 1	1.547966	0.040375	296,219	Down
2	Rab34	RAB34, member RAS oncogene family	1.510723	0.006158	360,571	Down
3	LOC680825	Hypothetical protein LOC680825	1.506099	0.022015		Down
4	Arhgef18	UNCHARACTERIZED LOC102551305; (TUTs) Chalmel, et. al	1.490087	0.038196		Down
5	Erp27	Endoplasmic reticulum protein 27	1.487307	0.000413	297,698	Down
6	Cxcl11	Chemokine (C-X-C motif) ligand 11	1.475777	0.028613	305,236	Down
7	Pate4	Prostate and testis expressed 4	1.465625	0.019817	363,041	Down
8	Mex3b	mex-3 RNA binding family member B	1.435734	0.039927	308,790	Down
9	Serpinb7	Serpin peptidase inhibitor, clade B (ovalbumin), member 7	1.423659	0.022143	117,092	Down
10	LOC103690171	Melanoma-associated antigen 1-like	1.400799	0.037697	103,690,171	Down
11	Fhl5	Four and a half LIM domains 5	− 1.51236	0.024828	297,954	Up
12	RT1-M6-2	RT1 class I, locus M6, gene 2	− 1.53651	0.028189	365,527	Up
13	Ahsp	Alpha hemoglobin stabilizing protein	− 1.54904	0.009705	293,522	Up
14	Tnfsf8	Tumor necrosis factor (ligand) superfamily, member 8	− 1.5581	0.040134	683,163	Up
15	Olr630	Olfactory receptor 630	− 1.56967	0.018284	405,947	Up
16	Spats2l	Spermatogenesis associated, serine-rich 2-like	− 1.5982	0.004577	316,426	Up
17	Mir351; Mir503	microRNA 351; microRNA 503	− 1.80708	0.004688	100,314,289;	Up
18	Neb	Nebulin [Source:RGD Symbol;Acc:1311134];	− 1.90567	0.008711		Up
19	Apold1	Apolipoprotein L domain containing 1	− 2.15289	0.035238	444,983	Up
20	Pla2g2a	Phospholipase A2, group IIA (platelets, synovial fluid)	− 3.14207	0.026596	29,692	Up

#### PCR validation of PLA2G2A and NXT1 expression and comparison with GEO datasets

**Fig. 7:**
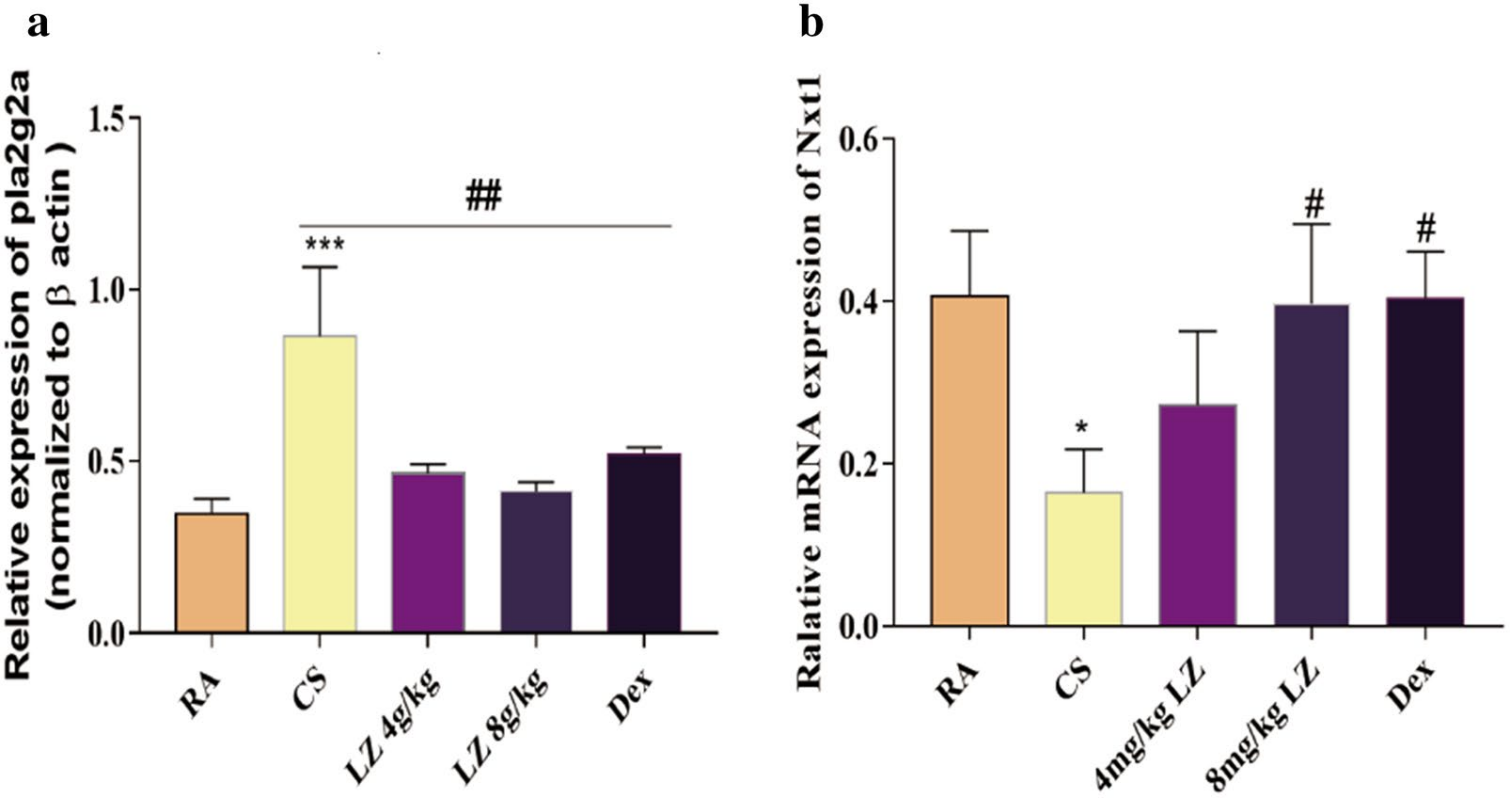
PCR analysis. **a** Relative expression of PLAG2A mRNA. **b** Relative expression of Nxt1 mRNA. Data expressed as means ± SD. Compared with control: *p < 0.05, **p < 0.01, ***p < 0.001 Compared with model: ^#^p < 0.05, ^##^p < 0.01, ^###^p < 0.001

To validate the gene expression datasets, we selected two genes which were strongly upregulated (NXT1 > 1.5 fold) and downregulated (PLA2G2A >− 3-fold) after Loki zupa treatment and determined gene expression by qPCR. Results showed more robust reduction of PLA2G2A expression and increased Nxt1 expression after Loki zupa treatment as compared to model group (p < 0.001, Fig. 7a, b). And these two genes were defined as LZ modulated “signature” genes and compared with the transcriptomic GEO data set (GSE1650) comprising gene array data from lung tissue from smoker with sever emphysema and non-smokers (Additional file 2: Figure S2). Results showed that Nxt1 was down regulated (p < 0.05) while there was upregulation trend in Pla2g2a expression in smoker with sever emphysema patients in different level as compared to non-smoker (Table 2). Interestingly, both microarray and qPCR results displayed these two genes expression level was reversed by LZ treatment in COPD rat lung tissue.

**Table 2:** NXT1 and Pla2g2a expression in transcriptomic GEO data set

Gene.symbol	Gene.title	LogFC	p value
NXT1	Nuclear transport factor 2 like export factor 1	− 0.7084152	0.00943915
PLA2G2A	Phospholipase A2 group IIA	0.6041078	0.2201457

### Effects of LZ on IL-6- Pla2g2a signaling in COPD rat lung tissue

**Fig. 8:**
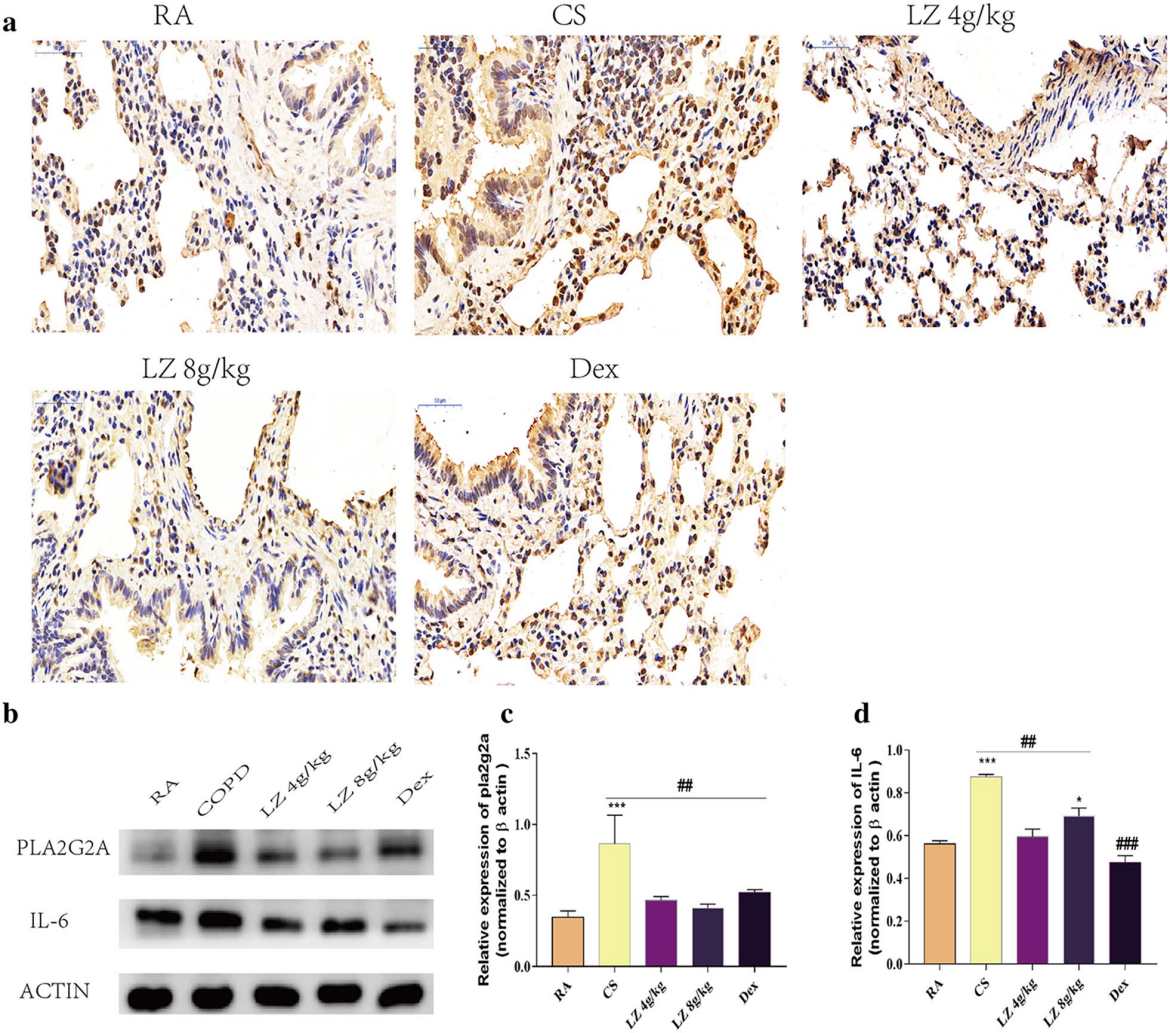
Effects of LZ on IL-6-PLAG2A signaling. **a** Immunohistochemistry analysis of PLA2G2A (200×); **b** Western blot images; **c** Relative expression of PLAG2A; **d** Relative expression of IL-6. Data expressed as means ± SD. Compared with control: *p < 0.05, **p < 0.01, ***p < 0.001 Compared with model: ^#^p < 0.05, ^##^p < 0.01, ^####^P < 0.001

Pla2g2a was top downregulated genes by LZ treatment, It was reported that IL-6 signaling amplified airway disease gene PLA2G2A expression and increase inflammatory responses in airway smooth muscle. Thus, we further assessed IL-6 and Pla2g2a protein expression in CS induced COPD rats by both western blot and immunohistochemistry analysis. There was a significant increase in both IL-6 and PLA2G2A protein expression after 24 week CS exposure, and this increasing trend was reversed by LZ treatment (Fig. 8b–d, p < 0.01). Immunohistochemistry analysis also showed increased expressions of Pla2g2a in CS group while there was a significant reduction after LZ treatment.

### Effects of LZ on protein expression of TGF-β, smad2/3, p-smad2/3 and collegen expression

**Fig. 9:**
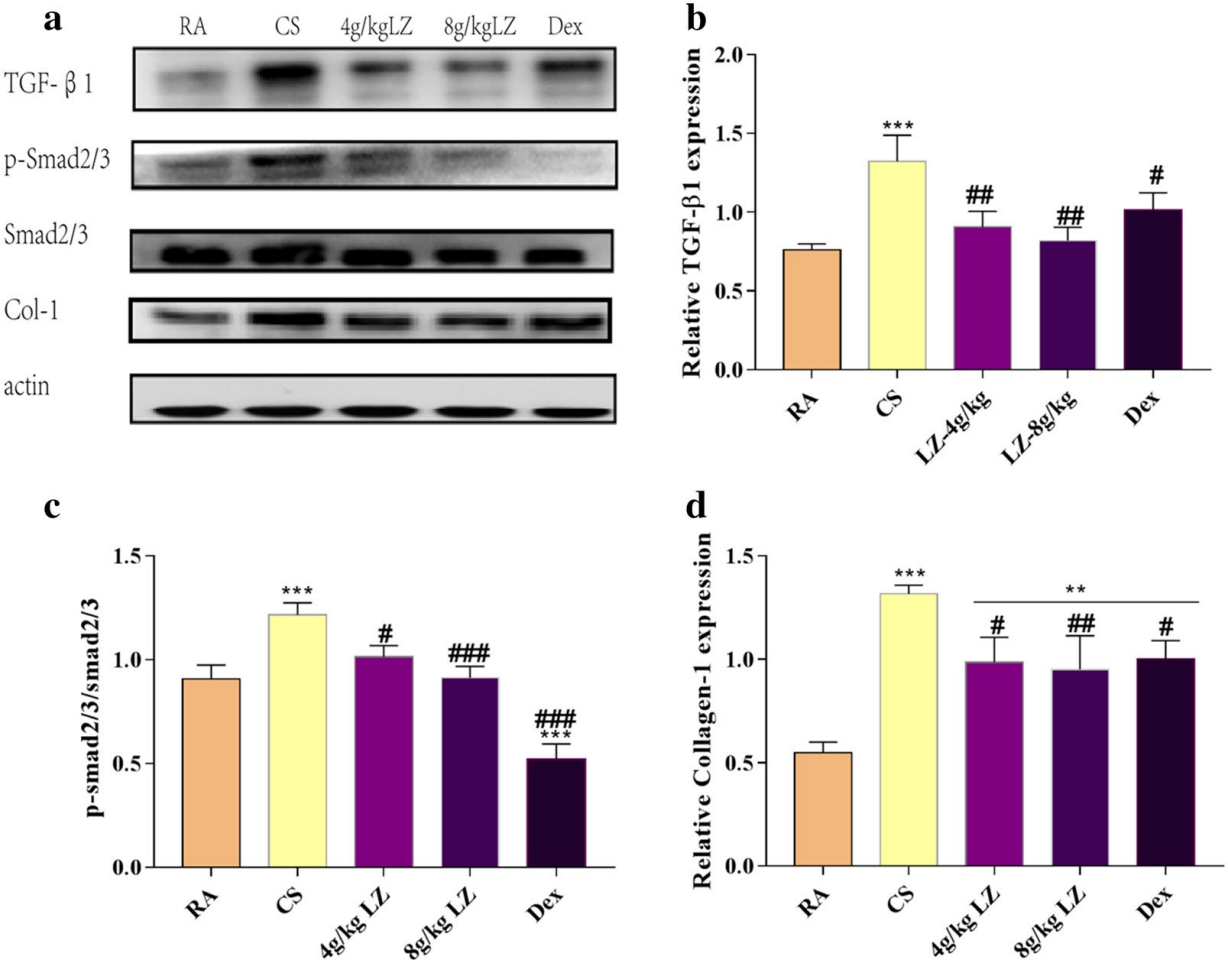
Effects of LZ on TGF-β signaling pathway. **a** Western blot images. **b** Relative expression of TGF-β 1; **c** relative expression of p-smad2/3/smad2/3; **d** relative expression of collgen-1 Protein. Data expressed as means ± SD. Compared with control: *p < 0.05, **p < 0.01, ***p < 0.001 Compared with model: ^#^p < 0.05, ^##^p < 0.01, ^####^p < 0.001

Microarray data analysis showed that LZ treatment also modulated extracellular space, and developmental process are also positively regulated. Collagen as the main extracellular matrix component, TGF-β1 is a protein related with developmental process. We found that Col-1 expression was elevated in CS exposure rats in comparison to those in RA rats(p < 0.001, Fig. 9a, d), however, rats in LZ group and Dex group significantly reduced col-1 expression as compared to CS group(p < 0.05, p < 0.01, respectively). Then we also measured upstream protein expression of Col-1. The alterations in the *TGF-β*/smad signaling pathway in pulmonary tissues was also determined using the Western blot analysis. The CS group rats showed increased expression of TGF*-β1* and p-smad2/3 in lung tissue as compared to the RA rats (p < 0.01, p < 0.001). Additionally, while significant reduction of TGF*-β1* and p-smad2/3 was observed after LZ and Dex treatments (p < 0.05, p < 0.01, respectively, Fig. 9a, d).

### Discussion

In this study, we explored the anti-airway inflammation and anti-remodeling effects of LZ in a cigarette smoke induced COPD rat model, which was likely achieved by modulating the IL-6/ PLA2G2A and TGF-β/smad pathway. As far as we know, this is the first report to demonstrate that Loki zupa could protect lungs from CS exposure and PLA2G2A gene role in COPD pathogenesis. Cigarette smoke induced rat models are widely used to explore disease pathogenesis and also used to screen potential therapies for COPD. There are two main characteristics of COPD: chronic bronchitis and destruction of lung parenchyma [25]. Pulmonary function is the gold standard for the diagnosis of COPD [26]. In this study, decline in lung function, pathological features such as small airway obstruction, infiltrated inflammatory cells in lung tissue, destruction of lung parenchyma, and increasing thickness of smooth muscle in bronchus in CS exposure groups proved that we successfully established a rat model of COPD.

Until now, the pathogenesis of COPD is poorly understood, however, it involves abnormal inflammatory and uncontrolled cellular responses to CS stimulation in the lung. Studies have shown that various inflammatory factors are increased in the lungs of patients with COPD, including networks of cytokines and chemokines that maintain inflammation and recruit circulating cells into the lungs [27]. IL-6 as an inflammatory cytokine which associated with the severity of acute COPD exacerbations and lung function decline [28]. IL-6 activates intracellular signaling pathways and induces gene expression and results in changes in human airway and act as a proinflammatory cytokines in COPD [29]. PLA2G2A which encodes the acute phase protein (sPLA2), is most highly expressed genes by IL-6 treatment in airway smooth muscle cell. It is also secreted by lung mast cells and macrophages and can be detected in BALF of patients with pneumonia [12, 30]. In pulmonary tissue, sPLA2 causes lung surfactant degradation, and promotes production of the precursor to inflammatory prostaglandins and the bronchoconstriction leukotrienes [31]. An in vivo and in vitro studies reported declined lung cancer cells after knock down of the PLA2G2A gene [32]. Interestingly. Our microarray, q-PCR, immunohistochemistry and western blot analysis showed that increased PLA2G2A expression in rat lung after 24 week CS exposure. And elevated IL-6 level in both blood serum and lung tissue. However, LZ treatment significantly reduced both IL-6 and PLA2G2A gene and protein expression in lung tissue. This may be the possible mechanism LZ contributes anti-airway inflammation role in COPD rats. Dex is one of the glucocorticoids inhibits the various inflammatory genes in COPD, by recruitment of histone deacetylase-2 to the activated inflammatory gene transcription complex and binding glucocorticoid receptors (GR) to coactivator molecules, Glucocorticoids also decline the stability of some pro-inflammatory mRNA species, including IL-6, TNF- α and IL-β, etc. [33–35]. IL-6 activates PLA2G2A gene expression and amplifies inflammatory responses. In the study we observed more robust decline of PLA2G2A expression in LZ group than dex group. Dex decreased PLA2G2A expression by blocking IL-6 expression. By contrast, our gene microarray and other results showed that LZ directly targets and significantly decreased Pla2g2a expression.

CS induced animal model of COPD shows elevated level of IL-6, TNF-α, IL- β, MMP-2, TGF-β and decreased IL-10 level in blood serum and BALF in different degrees [36–38]. In this study, Pro-inflammatory and anti-inflammatory cytokines had same expression trend with previous studies. However, As indicated in the blood serum, LZ-treated COPD rats restored pro-inflammatory and anti-inflammatory imbalance on different levels.

MMPs are also activated by smoking or oxidative stress [39], which are produced by a range of stromal cells and by two of the major inflammatory cells implicated in COPD – neutrophils and alveolar macrophages [40]. They inhibit the degradation of the extracellular matrix (ECM), whose changes in composition and quantity are associated with wall thickening [41]. In this study, we observed 8 g/kg LZ treatment promotes ECM degradation. Furthermore, inflammatory responses stimulate fibrotic responses by activating different signal pathways. Number of studies show increased collagen deposition in CS induced COPD rat models [42]. In this study, the CS group displayed increased collagen deposition and collagen-1 expression, evidenced by Mason's trichrome staining and Western Blot analysis. However, LZ-treated rats had markedly declined collagen deposition and collagen-1 expression in pulmonary tissue compared with that of CS exposed rats. These results indicate that LZ induced anti-airway remodeling effects in the CS induced COPD rat model.

One of the requirements of a successful COPD animal model is establishment of pathological changes including goblet epithelium cells metaplasia, evident epithelial cells injury of bronchi, mucus hypersecretion and cell blocking in bronchial lumen [43–45]. In addition, airway mucus hypersecretion is a cardinal feature of chronic obstructive pulmonary disease [46]. Excessive mucus production and secretion in proximal and in distal airways are associated with disabling symptoms (cough and sputum), declined lung function, exacerbations and death rate in patients’ with chronic obstructive pulmonary disease [47]. Present study results demonstrated that CS induced COPD rats exert excessive goblet cell metaplasia and had higher mucus secretion in contrast to normal group evidenced by PAS staining. Nonetheless, LZ treatment inhibited excessive goblet cell metaplasia and mucus hypersecretion.

As an chronic inflammatory disease, most interventions for COPD target inflammation. It was known that NF-κB is a cardinal molecule regulating inflammatory pathway and it was clear that CS exposure activates NF-κB pathways [48, 49]. Additionally, NF-κB also stimulates the expression of TGF-β1, an important proinflammatory cytokine with strong fibrotic effect. It activates TGF-β1/Smad2/3 signaling by binding with NF-κB at the combining site located on TGF-β1. It plays a vital role in inflammatory injury and repair especially in airway remodeling by activating Smad2/3, a downstream receptor kinase of TGF-β1 [50–52].

## Conclusion

Loki zupa treatment improves lung function in CS induced rat model of by reducing oxidative stress, inflammation and fibrotic responses. This mainly because forestalling the activation of the TFG-β-smad2/3 pathways and by blocking amplification of IL-6 /PLA2G2A signaling. And PLA2G2A is a significant gene associtated with COPD progression and can be a potential target for inhibiting the inflammatory responses in COPD. Therefore, this study would provide an experimental clue for future development and the clinical application of loki zupa on the treatment of COPD.

## Supplementary information

**Additional file 1: Figure S1.** Heatmap analysis of differentially expressed genes by Loki zupa.

**Additional file 2: Figure S2.** GEO Date sets from smoker with emphysema patients.

## Data Availability

The datasets used and/or analyzed in this study will be available with reasonable request.

## Abbreviations

COPD: Chronic obstructive pulmonary disease
LZ: Loki zuppa
FEV_0.1__s_: Forced expiratory volume in 0.1 s
FVC: Forced vital capacity
MMEF: Maximal midexpiratory flow curve
PEF: Peak expiratory flow
IL-6: Interleukin-6
IL-1β: Interleukin-1β
TNF-α: Tumor necrosis factor- α
IL-10: Interleukin-10
MMP-9: Matrix metalloproteinase-9
TGF-β1: Transforming growth factor beta-β1
MDA: Malondialdehyde
SOD: Superoxide dismutase
