# Supplementary figures and images for: Loki zupa alleviates inflammatory and fibrotic responses in cigarette smoke induced rat model of chronic obstructive pulmonary disease
Source: Chin Med. 2020 Aug 31;15:92. doi: 10.1186/s13020-020-00373-3 (PMC7457355; doi:10.1186/s13020-020-00373-3)

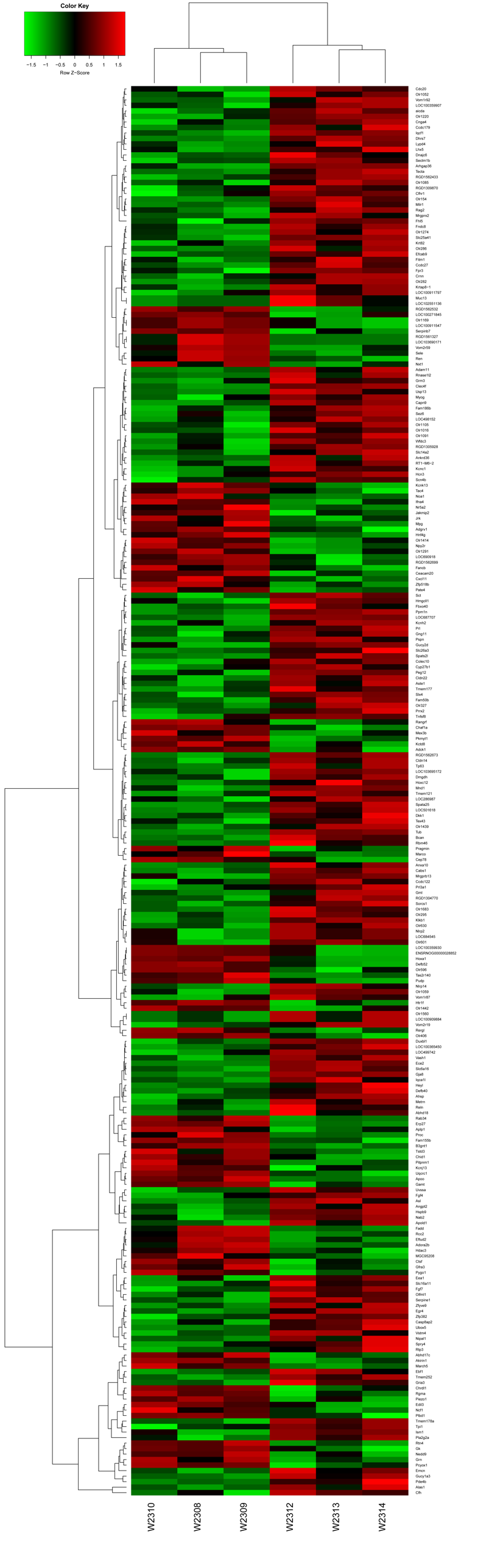

Supplement: Supplementary file 1 — Additional file 1: Figure S1. Heatmap analysis of differentially expressed genes by Loki zupa. [file 13020_2020_373_MOESM1_ESM.tif]

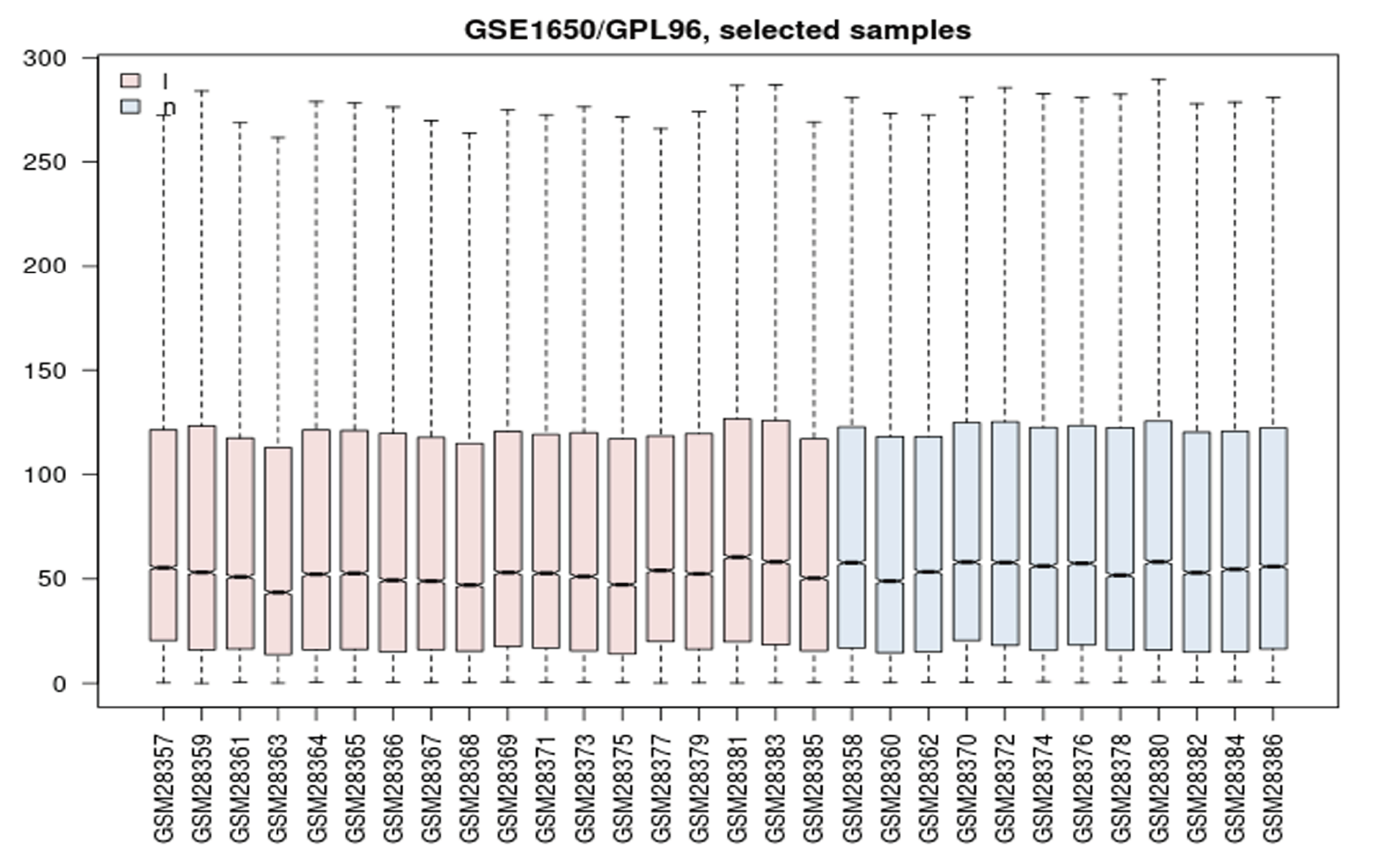

Supplement: Supplementary file 2 — Additional file 2: Figure S2. GEO Date sets from smoker with emphysema patients. [file 13020_2020_373_MOESM2_ESM.tif]
